# Long term cognitive dysfunction among critical care survivors: associated factors and quality of life—a multicenter cohort study

**DOI:** 10.1186/s13613-024-01335-w

**Published:** 2024-07-29

**Authors:** Isabel Jesus Pereira, Mariana Santos, Daniel Sganzerla, Caroline Cabral Robinson, Denise de Souza, Renata Kochhann, Maicon Falavigna, Luis Azevedo, Fernando Bozza, Tarek Sharshar, Regis Goulart Rosa, Cristina Granja, Cassiano Teixeira

**Affiliations:** 1 Department of Surgery and Physiology, Faculty of Medicine, University of Porto, Porto, Portugal; 2 SIM-FMUP-Simulation Center, Faculty of Medicine, University of Porto, Porto, Portugal; 3 CriticalMed–Critical Care & Emergency Medicine, CINTESIS-Center for Health Technology and Services Research, University of Porto, Porto, Portugal; 4 Intensive Care Department, Centro Hospitalar de Gaia/Espinho, Vila Nova de Gaia, Portugal; 5 MEDCIDS–Medicina da Comunidade, Informação E Decisão Em Saúde, Department of Community Medicine, Information and Health Decision Sciences, Faculty of Medicine, University of Porto, Porto, Portugal; 6 CINTESIS@RISE–Center for Health Technology and Services Research (CINTESIS) & Health Research Network Associated Laboratory (RISE), University of Porto, Porto, Portugal; 7 Research Unit, INOVA Medical, Porto Alegre, RS Brazil; 8 Research Projects Office, Hospital Moinhos de Vento, Porto Alegre, Brazil; 9 Institute for Health Technology Assessment, Postgraduate Program in Epidemiology, Universidade Federal Do Rio Grande Do Sul (UFRGS), Porto Alegre, Brazil; 10 Postgraduate Program in Epidemiology, UFRGS, Porto Alegre, Brazil; 11 Department of Critical Care, D’Or Institute for Research and Education, Rio de Janeiro, RJ Brazil; 12 Anesthesia and Intensive Care Department, Institute of Psychiatry, GHU Paris Psychiatrie Et NeurosciencesSainte-Anne HospitalNeurosciences of Paris, INSERM U1266, Université Paris Cité, Pole Neuro, ParisParis, France; 13 Department of Internal Medicine, Hospital Moinhos de Vento, Porto Alegre, RS Brazil; 14 Brazilian Research in Intensive Care Network, São Paulo, SP Brazil; 15 Anaesthesiology Department, University Hospital Center São João, Porto, Portugal; 16 Intensive Care Department, Hospital de Clínicas de Porto Alegre, Porto Alegre, RS Brazil; 17 UFCSPA Medical School, Porto Alegre, RS Brazil

**Keywords:** Long-term cognitive dysfunction, Critical care survivors, Follow-up, Cognitive reserve, Health-related quality of life, Delirium

## Abstract

**Objectives:**

To identify the prevalence and associated factors of cognitive dysfunction, 1 year after ICU discharge, among adult patients, and it´s relation with quality of life.

**Methods:**

Multicenter, prospective cohort study including ICUs of 10 tertiary hospitals in Brazil, between May 2014 and December 2018. The patients included were 452 adult ICU survivors (median age 60; 47.6% women) with an ICU stay greater than 72 h.

**Results:**

At 12 months after ICU discharge, a Montreal Cognitive Assessment (tMOCA) telephone score of less than 12 was defined as cognitive dysfunction. At 12 months, of the 452 ICU survivors who completed the cognitive evaluation 216 (47.8%) had cognitive dysfunction.

In multivariable analyses, the factors associated with long-term (1-year) cognitive dysfunction were older age (Prevalence Ratio–PR = 1.44, P < 0.001), absence of higher education (PR = 2.81, P = 0.005), higher comorbidities on admission (PR = 1.089; P = 0.004) and delirium (PR = 1.13, P < 0.001). Health-related Quality of life (HRQoL), assessed by the mental and physical dimensions of the SF-12v2, was significantly better in patients without cognitive dysfunction (Mental SF-12v2 Mean difference = 2.54; CI 95%, − 4.80/− 0.28; p = 0.028 and Physical SF-12v2 Mean difference = − 2.85; CI 95%, − 5.20/− 0.50; P = 0.018).

**Conclusions:**

Delirium was found to be the main modifiable predictor of long-term cognitive dysfunction in ICU survivors. Higher education consistently reduced the probability of having long-term cognitive dysfunction. Cognitive dysfunction significantly influenced patients’ quality of life, leading us to emphasize the importance of cognitive reserve for long-term prognosis after ICU discharge.

**Supplementary Information:**

The online version contains supplementary material available at 10.1186/s13613-024-01335-w.

## Background

Brain dysfunction related with critical illness [1] encompasses a broad spectrum of pathology from acute dysfunction as delirium, to long-term cognitive dysfunction [2]. Surviving ICU carries a burden related with brain dysfunction and poor quality of life [3–5], but despite the increasing amount of evidence, cognitive dysfunction natural history remains unclear.

Cognitive dysfunction develops through a complex interaction between patient’s baseline vulnerability (i.e. age, genetic predisposition, preexisting cognitive dysfunction) [6, 7] and precipitating factors (i.e. delirium, sepsis, surgery/anesthetics, metabolic derangement). Its prevalence is highly heterogeneous and influenced by the cognitive assessment test used, the time of analysis and also by the population studied (disease specific or mixed populations of medical and surgical ICU patients) [8] Cognitive dysfunction assessed early after hospital discharge has been described to affect up to 100% of the ICU survivors [9], and, even though cognitive dysfunction prevalence decreases over time after ICU discharge, long-term follow-up reveals high levels of long-term cognitive impairment several years after ICU discharge, in some series affecting 80% of the ICU survivors after one year and 45% after 2 years [8, 10].

Worse cognition has been described to be associated with worse quality of life scores [4] but the attributable impact of pre-ICU health, critical illness, and post-ICU disabilities on long-term cognitive function is not clear, especially in a resource-limited context like Brazil.

Early identification of patients at risk and the promotion of interventions on the associated factors may eventually improve outcome by reducing both acute and long-term cognitive dysfunction, ultimately improving quality of life.

In this study, the authors aim to investigate the prevalence of cognitive dysfunction 12 months after ICU discharge. The second objective is to identify factors associated with cognitive dysfunction 12 months after ICU discharge, including health related quality of life.

## Methods

### Study design

The present study is part of the Quality of life after intensive care unit: a multicenter cohort study for assessment of long-term outcomes among ICU survivors in Brazil. The study protocol has been published previously [11–13] The study was conducted from May 2014 to December 2018 in 10 Brazilian tertiary hospitals. Patients were recruited while still in the hospital and followed-up by telephone interviews at 3, 6 and 12 months after ICU discharge [12].

The study was planned under the Brazilian National Health Council Resolution no. 466/12 and approved by the research ethics committees of all participating centers. Informed consent was obtained from all study participants or their legal representatives [12].

### Participants

Patients over 18 years old with an ICU stay exceeding 72 h for medical or emergency surgery or 120 h for elective surgery, were consecutively screened for eligibility. Patients were included in the analysis if cognitive follow-up at 12 months was successful. Exclusion criteria were: previous dementia diagnosis, transfer between hospitals; direct discharge home from ICU; < 24 h ICU readmission after discharge; absence of proxy for patients with communication difficulties; impossibility of assessing the patient during the first 5 days after ICU discharge, refusal or withdrawal of agreement to participate; previous enrollment in the study; no available telephone contact, failure to complete one year follow-up [12].

### Associated factors

When considering associated factors for cognitive dysfunction, 5 sets of variables were evaluated:

1. Sociodemographic characteristics: age, sex, educational attainment, household income;
2. Health state 3 months before ICU admission: physical functional status; comorbidities; lifetime history of anxiety or depression;
3. Acute illness characteristics: ICU admission type, risk of death at ICU admission, presence of sepsis or ARDS, organ dysfunction during ICU stay, ICU acquired infections, length of ICU and hospital stay;
4. ICU discharge status: muscular strength, symptoms of anxiety and depression, cognitive function;
5. Status 12 months after ICU discharge: vital status; HRQoL.

Independent variables (1 to 4) were collected using structured face-to-face interviews, physical examination, and retrospective review of medical records performed at the moment of patient enrollment (24–120 h after ICU discharge) and during telephone interviews (variables 5).

Physical functional status was assessed by Barthel index [14], where physical dependence was defined as a score of less than or equal to 75 [15, 16]. Comorbidities were collected using the Charlson Comorbidity Index [17] dichotomized as low (score 0 or 1) or high comorbidity (≥ 2). The risk of death at ICU admission, was derived from the Acute Physiology and Chronic Health Evaluation II [18] or the Simplified Acute Physiology Score 3 [19]. Sepsis and acute respiratory distress syndrome were defined according to the sepsis-II [20] and Berlin [21] definitions, respectively. Organ dysfunction was defined as the presence of invasive mechanical ventilation, vasopressor, renal replacement therapy (except for patients under chronic dialysis treatment), parenteral nutrition, blood or blood products transfusion, and delirium (measured according the Confusion Assessment Method for the ICU [22]). ICU-acquired infections were defined, by chart review, as pneumonia, bloodstream or urinary tract infection occurring > 48 h of ICU admission according to the European Centre for Disease Prevention and Control criteria [23]. Muscular strength was assessed using the Medical Research Council Scale [24], with a cut off of < 48 for ICU acquired weakness (ICUAW) [25]. Anxiety and depression were assessed using the Hospital Anxiety and Depression Scale [23]. After ICU discharge, the Mini Mental State Examination (MMSE) [26] was applied. No higher education was defined as not holding a university degree. Some continuous variables were categorized using predefined relevant cut-off points to facilitate interpretation.

### Outcomes and follow-up

Researchers, not associated with patient care, assessed outcomes using a structured telephone interviews 3, 6 and 12 months after ICU discharge within a 30-day window (15 days before and 15 days after due date). Patients were categorized as follow-up losses after 10 unsuccessful attempts of telephone contact, at different times on several days within the window.

Family members were allowed to answer objective questions when patients lacked adequate physical or cognitive conditions. For subjective outcomes, like cognition or health related quality of life, family members were not allowed to interfere [12].

#### Cognitive function

To establish cognitive dysfunction, patients underwent a comprehensive neuropsychological evaluation at 12 months after ICU discharge. Immediately after ICU discharge was applied the Mini Mental State Examination (MMSE) [26] and twelve months after ICU discharge, cognitive function was assessed using the Brazilian version validated for telephone administration of the Montreal Cognitive Assessment (tMoCA) [27]. In tMoCA, the domains analyzed are short-term memory; executive function; attention, concentration, and working memory; language; orientation to time and place. It enables subject evaluation for mild cognitive impairment, irrespective of etiology. tMoCA scores range from 0 to 22; higher scores indicate better cognitive status. There is no specific data regarding tMoCa average score for cognitive impairment in ICU patients. In other populations, the MoCa average score for mild cognitive impairment is described to be 22, and 17 for moderate/severe cognitive impairment. In the literature, it has been described a tMoCA score of 12 as equivalent to a MoCA Full score of 17 [28, 29]. For clinical relevance, in the trade-off between sensitivity Vs specificity, and in order to identify the more severe patients, a tMoCA score of 12 was used as a cut-off for moderate/severe cognitive impairment. For reference, a tMoCa score of 16 was used as a cut off for mild cognitive impairment.

#### Health-related quality of life

The HRQoL was assessed at 12 months using the Short-Form Health Survey version 2 (SF-12v2) [30] The SF-12v2 addresses HRQoL in eight domains: general health, physical functioning, physical role function, bodily pain, vitality, emotional role function, mental health, and social functioning. These 8 domains are then summarized in 2 dimensions, physical and mental, and each domain scores ranges from 0 to 100, where higher scores indicate better HRQoL. A score of 42 or less on the mental SF-12v2 may be indicative of "clinical depression," whereas a score of 50 or less on the physical SF-12v2 has been recommended as a cut-off to indicate a physical condition [31].

### Statistical analysis

Continuous variables were registered as mean and standard deviation or median and interquartile range as appropriate. Categorical variables were expressed as absolute and relative frequencies. Associated factors for 12-month cognitive dysfunction were based on premorbid condition, during ICU stay, and in the immediate post-discharge period. They were assessed by calculating Prevalence Ratio (PR) as association measures, using Generalized Estimating Equations (GEE) Poisson models, in order to adjust for the effect of patients’ clustering within the 10 different centers/hospitals participating in the study. In the GEE models, a Poisson distribution was used for the binary response variable (primary outcome – presence of cognitive dysfunction), we assumed an exchangeable covariance matrix, and robust sandwich variance estimators were used to estimate the model coefficients. In this context, appropriately corrected robust variance estimators are essential to overcome the possible misspecification of the variance of Poisson distributions and of the working covariance matrix [32, 33]. Variables with P-value less than 0.20 were considered in the multivariable model and a stepwise backward selection method was used for variable selection.

In order to assess survival bias (influence of patients’ survival on the results), a sensitivity analysis was performed). In this analysis we also included patients who died during the 12 months follow-up by giving them a MoCA score of zero; thus, changing the primary outcome to a composite outcome of death or cognitive dysfunction.

All analyzes were conducted in the R software, version 4.2.2. A significance level of 5% was considered.

## Results

Among the 1,616 patients included in the Post-ICU Quality of Life Project [12], 98 patients were excluded due to previous dementia; of the 1108 patients alive after 12 months of follow-up, 656 patients were excluded due to lack of cognitive assessment (Supplemental Figure 1 and Supplemental Table S1). A total of 452 patients were analyzed (Table ﻿1). The characteristics of the 10 participating hospitals that recruited patients are shown in Supplemental Table S2.

### Characteristics of the Cohort

In Table 1 we describe the characteristics of the 452 participants who completed the 12 months cognitive assessment. The median age was 60 years and 47.6% were women. Only 28.2% (n = 127/450) of the patients had higher education. Half the patients had high comorbidity (46.2%); 17.1% had past history of depression and 19,5% of anxiety. Medical condition accounted for 67.7% of all admissions. The need for invasive mechanical ventilation and vasopressor were the most frequent organ dysfunction [47.6% and 52% respectively]. Delirium was present in 19.9% of the patients. The median ICU and hospital length of stay (LOS) was 6 (IQR 4–10) and 21 (IQR 13–36) days. Early after ICU discharge, 39.1% of the patients presented anxiety symptoms; 21.6% depression symptoms; 32.5% cognitive dysfunction and 21.1% had ICUAW.

**Table 1:** Characteristics of patients

Characteristics	Total
**Sociodemographic characteristics**	
Age, years—median (IQR)	60 (47.8–68.2)
Age ≥ 65 years—no./total no. (%)	166/452 (36.7)
Female sex—no./total no. (%)	215/452 (47.6)
Educational attainment, years—median (IQR)	11 (8–16)
No Higher education—no./total no. (%)	323/450 (71.8)
Monthly per capita household income^a^, USD—median (IQR)	671.7 (403–1641.2)
**State of health before admission to the ICU**	
Charlson comorbidity index—median (IQR)	1 (0–3)
Charlson comorbidity index ≥ 2—no./total no. (%)	209/452 (46.2)
History of depression—no./total no. (%)	77/449 (17.1)
History of anxiety—no./total no. (%)	88/451 (19.5)
Barthel Index—median (IQR)	100 (95–100)
[0–25]-Total	2/451 (0.4)
(25–50]-Severe	7/451 (1.6)
(50–75]-Moderate	13/451 (2.9)
(75–99]-Mild	111/451 (24.6)
'100—Independent	318/451 (70.5)
**Characteristics of acute critical illness**	
ICU Admission type	
Medical—no./total no. (%)	306/452 (67.7)
Surgical, elective—no./total no. (%)	89/452 (19.7)
Surgical, emergency—no./total no. (%)	57/452 (12.6)
Risk of death at ICU admission^b^, %—median (IQR)	14.6 (8.7–26.2)
Severe sepsis or septic shock at ICU admission^c^—no./total no. (%)	152/452 (33.6)
ARDS at ICU admission^d^– no./total no. (%)	38/452 (8.4)
Organ dysfunction^e^ during ICU stay	
Number of organ dysfunctions—median (IQR)	1 (0–2)
Need of invasive mechanical ventilation—no./total no. (%)	215/452 (47.6)
Days of invasive mechanical ventilation—median (IQR)	0 (0–3)
Need of vasopressor—no./total no. (%)	235/452 (52)
Need of renal replacement therapy—no./total no. (%)	46/452 (10.2)
Need of parenteral nutrition—no./total no. (%)	28/452 (6.2)
Need of blood or blood products transfusion—no./total no. (%)	66/452 (14.6)
Delirium—no./total no. (%)	90/452 (19.9)
ICU-acquired infection^f^—no./total no. (%)	48/452 (10.6)
ICU length of stay, days, median (IQR)	6 (4–10)
Hospital length of stay, days, median (IQR)	21 (13–36)
**State of health immediately after ICU discharge (24 to 120 h)**	
Respondents—HADS—no./total no. (%)	412/452 (91.2)
HADS-a—median (IQR)	6 (3–9.2)
Anxiety symptoms^g^ (HADSa > 7)—no./total no. (%)	161/412 (39.1)
HADS-d—median (IQR)	4 (2–7)
Depression symptoms^h^ (HADSd > 7)—no./total no. (%)	89/412 (21.6)
Respondents—MMSE^i^- no./total no. (%)	363/452 (80.3)
MMSE score—median (IQR)	25 (23–27)
Cognitive dysfunction—no./total no. (%)	118/363 (32.5)
Respondents—MRC—no./total no. (%)	322/452 (71.2)
MRC—median (IQR)	55 (48–60)
Muscular weakness (MCR < 48)—no./total no. (%)	68/322 (21.1)

IQR, interquartile range (p25; p75); ICU, intensive care unit; ARDS, cute respiratory distress syndrome; HADS, hospital anxiety and depression scale; MMSE, mini-mental state evaluation; MRC, medical research council scale

^a^USD, United States dollar using the purchasing power parity conversion (BRL to USD);

^b^Predicted risk of hospital death derived from the Acute Physiology and Chronic Health Evaluation-II (APACHE-II) or the Simplified Acute Physiology Score-3 (SAPS-3);

^c^According to the sepsis-II criteria;

^d^According to Berlin criteria;

^e^Defined as the presence of any of the following during ICU stay: need of invasive mechanical ventilation, vasopressor, renal replacement therapy (except for patients under chronic dialysis treatment), parenteral nutrition, blood or blood products transfusion; and delirium (measured according the Confusion Assessment Method for the ICU);

^f^Defined as pneumonia, bloodstream or urinary tract infection occurring > 48 h of ICU admission according to the European Centre for Disease Prevention and Control criteria

^g^Anxiety Hospital Anxiety and Depression Scale anxiety subscale score > 7

^h^Anxiety Hospital Anxiety and Depression Scale depression subscale score > 7

^I^Mini Mental State Examination ≤ 21 if 4 years or less of educational attainment, or ≤ 24 if > 4 years of educational attainment

### 12 Months prevalence of cognitive dysfunction and associated factors

**Fig. 1:**
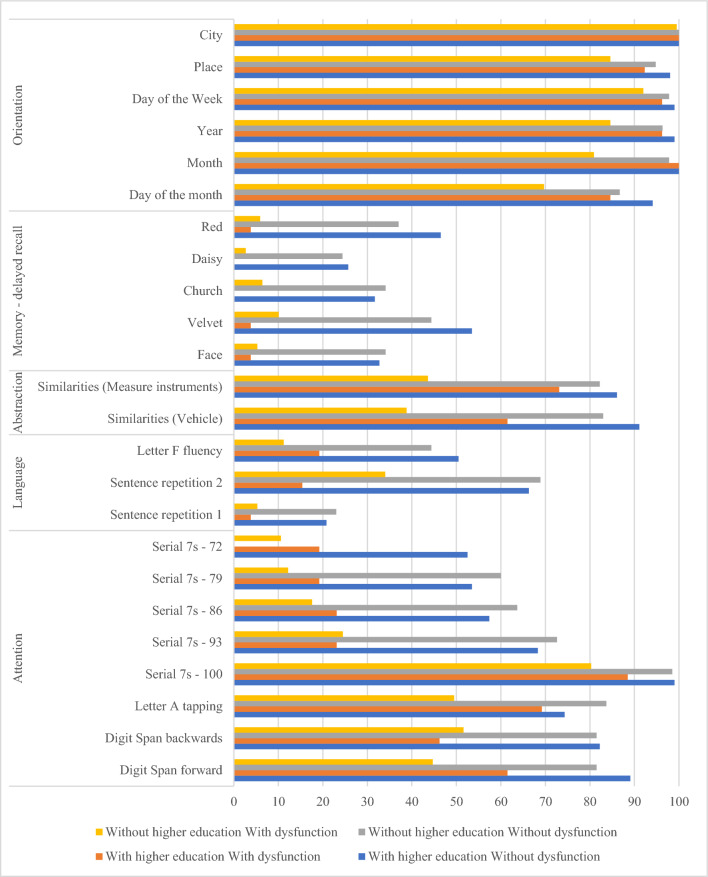
Proportion of correct tMOCA answers by subdomain, presence of cognitive dysfunction and higher education

Twelve months after ICU discharge, of the 452 patients who were able to complete cognitive evaluation, 351 (77.7%) had some degree of cognitive dysfunction (tMOCA < 16) of whom 216 (47.8%) had moderate to severe cognitive dysfunction (tMOCA < 12). Up to different extent, all the subdomains of tMOCA were affected both in patients with or without cognitive dysfunction. In patients with cognitive dysfunction the least affected was orientation (correct response rate over 70%) and the most severely affected were delayed recall (incorrect response rate over 90%) and serial subtraction (incorrect response rate over 75%). Abstraction accounted for the higher difference in correct responses in patients with or without cognitive dysfunction. (Fig. 1).

In the univariable analysis (Table 2), factors identified to be associated with higher risk for cognitive dysfunction at 1 year are as follows: increasing age (PR 1.02; P < 0.001), no higher education (PR 2.07, P < 0.001), and pre-ICU high comorbidity (PR 1.2, P < 0.001). On the other hand, educational attainment (PR 0.92, P < 0.001) and household income (independent of number of minimum wages considered) were considered protective.

**Table 2:** Univariable analysis of factors associated with cognitive dysfunction

	No cognitive dysfunction	Cognitive dysfunction	Prevalence ratio (PR) (95% CI)	*P value*
**Sociodemographic characteristics**				
Age, years—median (IQR)	57 (41–66.2)	62 (53–71)	1.02 (1.01–1.02)	< 0.001
Age ≥ 65 years—no./total no. (%)	73/236 (30.9)	93/216 (43.1)	1.45 (1.19–1.76)	< 0.001
Female sex—no./total no. (%)	118/236 (50)	97/216 (44.9)	0.92 (0.80–1.05)	0.229
Educational attainment, years—median (IQR)	11 (11–16)	8 (5–11)	0.92 (0.90–0.95)	< 0.001
No Higher education—no./total no. (%)	135/236 (57.2)	188/214 (87.9)	2.07 (1.71–2.51)	0.001
Household income^a^				< 0.001
1	59/152 (38.8)	109/153 (71.2)	-	-
2 to 5	75/152 (49.3)	35/153 (22.9)	0.61 (0.5–0.74)	< 0.001
More than 5	18/152 (11.8)	9/153 (5.9)	0.68 (0.47–0.98)	0.039
**State of health before admission to the ICU**				
Charlson comorbidity index—median (IQR)	1 (0–2)	2 (0–3)	1.05 (1.03–1.07)	< 0.001
Charlson comorbidity index ≥ 2—no./total no. (%)	93/236 (39.4)	116/216 (53.7)	1.2 (1.13–1.27)	< 0.001
History of depression—no./total no. (%)	42/235 (17.9)	35/214 (16.4)	0.94 (0.62–1.44)	0.784
History of anxiety—no./total no. (%)	51/236 (21.6)	37/215 (17.2)	0.84 (0.76–0.92)	0.001
Barthel Index—median (IQR)	100 (95–100)	100 (95–100)	1 (0.99—1.01)	0.902
[0–25]-Total	1/235 (0.4)	1/216 (0.5)		
(25–50]-Severe	4/235 (1.7)	3/216 (1.4)		
(50–75]-Moderate	7/235 (3)	6/216 (2.8)		
(75–99]-Mild	54/235 (23)	57/216 (26.4)		
'100—Independent	169/235 (71.9)	149/216 (69)		
**Characteristics of acute critical illness**				
ICU Admission type			1.01 (0.91–1.12)	0.836
Medical—no./total no. (%)	160/236 (67.8)	146/216 (67.6)		
Surgical, elective—no./total no. (%)	46/236 (19.5)	43/216 (19.9)		
Surgical, emergency—no./total no. (%)	30/236 (12.7)	27/216 (12.5)		
Risk of death at ICU admission^b^, %—median (IQR)	14.6 (8.7–23.5)	15.6 (11.3–33.1)	1.11 (0.84–1.46)	0.486
Severe sepsis/septic shock at ICU admission^c^ no./total no. (%)	74/236 (31.4)	78/216 (36.1)	0.92 (0.76–1.11)	0.363
ARDS at ICU admission^d^—no./total no. (%)	22/236 (9.3)	16/216 (7.4)	0.77 (0.52–1.13)	0.183
Organ dysfunction^e^ during ICU stay				
Number of organ dysfunctions—median (IQR)	1 (0–2)	1 (0–2)	0.97 (0.84–1.11)	0.646
Need of invasive mechanical ventilation—no./total no. (%)	117/236 (49.6)	98/216 (45.4)	0.83 (0.72–0.96)	0.01
Days of invasive mechanical ventilation—median (IQR)	0 (0–4)	0 (0–2.2)	0.99 (0.96–1.02)	0.544
Need of vasopressor—no./total no. (%)	128/236 (54.2)	107/216 (49.5)	0.87 (0.75–1)	0.043
Need of renal replacement therapy—no./total no. (%)	25/236 (10.6)	21/216 (9.7)	0.89 (0.77–1.02)	0.089
Need of parenteral nutrition—no./total no. (%)	11/236 (4.7)	17/216 (7.9)	1.3 (1.07–1.58)	0.008
Need of blood or blood products transfusion—no./total no. (%)	37/236 (15.7)	29/216 (13.4)	0.82 (0.7–0.97)	0.021
Delirium—no./total no. (%)	38/236 (16.1)	52/216 (24.1)	1.11 (1.03–1.19)	0.005
ICU-acquired infection^f^—no./total no. (%)	29/236 (12.3)	19/216 (8.8)	0.69 (0.5–0.95)	0.021
ICU length of stay, days, median (IQR)	7 (4–11)	6 (4–9)	0.98 (0.97–1)	0.116
Hospital length of stay, days, median (IQR)	21 (14–38.2)	19 (13–34.2)	0.99 (0.99–1)	0.046
**State of health immediately after ICU discharge (24 to 120 h)**				
Respondents—HADS—no./total no. (%)	214/236 (90.7)	198/216 (91.7)	1.07 (0.92–1.25)	0.388
HADS-a—median (IQR)	5.5 (3–9)	7 (3–10)	1.02 (1.01–1.02)	< 0.001
Anxiety symptoms^g^ (HADSa > 7)—no./total no. (%)	70/214 (32.7)	91/198 (46)	1.19 (1.13–1.26)	< 0.001
HADS-d—median (IQR)	4 (2–7)	5 (3–7)	1.02 (0.99–1.05)	0.256
Depression symptoms^h^ (HADSd > 7)—no./total no. (%)	42/214 (19.6)	47/198 (23.7)	1.08 (0.83–1.41)	0.552
Respondents—MMSE^i^- no./total no. (%)	187/236 (79.2)	176/216 (81.5)	1.02 (0.94–1.12)	0.583
MMSE score—median (IQR)	27 (24–29)	24 (22–26)	0.95 (0.93–0.97)	< 0.001
Cognitive dysfunction—no./total no. (%)	48/187 (25.7)	70/176 (39.8)	1.27 (1.14–1.43)	< 0.001
Respondents—MRC—no./total no. (%)	158/236 (66.9)	164/216 (75.9)	1.02 (0.91–1.16)	0.703
MRC—median (IQR)	55.5 (48–60)	54.5 (48–60)	1 (0.99–1.01)	0.794
Muscular weakness (MCR < 48)—no./total no. (%)	34/158 (21.5)	34/164 (20.7)	0.96 (0.76–1.2)	0.718

IQR, interquartile range (p25; p75); ICU, intensive care unit; ARDS, cute respiratory distress syndrome; HADS, hospital anxiety and depression scale; MMSE, mini-mental state evaluation; MRC, medical research council scale

^a^Number of minimum wages;

^b^Predicted risk of hospital death derived from the Acute Physiology and Chronic Health Evaluation-II (APACHE-II) or the Simplified Acute Physiology Score-3 (SAPS-3);

^c^According to the sepsis-II criteria;

^d^According to Berlin criteria;

^e^Defined as the presence of any of the following during ICU stay: need of invasive mechanical ventilation, vasopressor, renal replacement therapy (except for patients under chronic dialysis treatment), parenteral nutrition, blood or blood products transfusion; and delirium (measured according the Confusion Assessment Method for the ICU);

^f^Defined as pneumonia, bloodstream or urinary tract infection occurring > 48 h of ICU admission according to the European Centre for Disease Prevention and Control criteria

^g^Anxiety Hospital Anxiety and Depression Scale anxiety subscale score > 7

^h^Anxiety Hospital Anxiety and Depression Scale depression subscale score > 7

^I^Mini Mental State Examination ≤ 21 if 4 years or less of educational attainment, or ≤ 24 if > 4 years of educational attainment

When considering organ dysfunction only the need of parenteral nutrition (PR 1.3, P = 0.008) and delirium (PR 1.11, P = 0.005) were significant for increased risk of cognitive dysfunction. On the other hand, invasive mechanical ventilation (PR 0.83, P = 0.01); vasopressor use (PR 0.87, P = 0.043) and blood products use (PR 0.82, P = 0.021) appeared to be protective against cognitive dysfunction. Also, the presence of ICU-acquired infection (PR 0.69, P = 0.021) and hospital LOS appear to be protective towards cognitive dysfunction (0.99, p = 0.046).

Contrary to anxiety after ICU discharge (PR 1.19, P < 0.001), past history of anxiety (PR 0.84; p = 0.001) appear to be protective towards cognitive dysfunction.

Cognitive dysfunction early after ICU discharge (PR 1.27, P < 0.001) was positively associated with long-term cognitive dysfunction, unlike muscular weakness (PR 0.96, p = 0.718).

Table 3 shows the multivariable analysis of factors independently associated with cognitive dysfunction. Higher education was consistently protective against long-term cognitive dysfunction both in univariate (no Higher education: PR 2.07, P < 0.001) and in multivariable analysis (no Higher education: PR 1.98, p = 0.005). Older age (PR 1.44, P < 0.001), delirium (PR 1.13, p < 0.001) and pre-ICU high comorbidity (PR 1.09, p = 0.004) were also associated with the probability of having long-term cognitive dysfunction.

**Table 3:** Multivariable analysis of risk factors associated with cognitive dysfunction

	Univariable		Multivariable	
	Prevalence ratio (PR) (95% CI)	*P value*	Prevalence ratio (PR) (95% CI)	*P value*
Age ≥ 65 years	1.45 (1.19–1.76)	< 0.001	1.44 (1.18–1.75)	< 0.001
Female sex	0.92 (0.80–1.05)	0.229	0.97 (0.87–1.07)	0.496
No Higher education	2.07 (1.71–2.51)	< 0.001	1.98 (1.7–2.3)	< 0.001
Charlson comorbidity index ≥ 2	1.20 (1.13–1.27)	< 0.001	1.09 (1.03–1.15)	0.004
Risk of death at ICU admission	1.11 (0.84–1.46)	0.486	1.002 (1–1.01)	0.116
Delirium^a^	1.11 (1.03–1.19)	0.005	1.13 (1.06–1.21)	< 0.001

IQR, interquartile range (p25; p75); ICU, intensive care unit

^a^Measured according the Confusion Assessment Method for the ICU

The sensitivity analysis, aimed at assessing the risk of survival bias, showed similar results to those of the main analyses (Supplemental Table S6) except for the fact that functional dependence and muscular strength now appear associated with cognitive dysfunction, and de novo also the risk of death at ICU admission. Delirium maintained its association with cognitive dysfunction, while ARDS at ICU appears to be protective against cognitive dysfunction.

### Health-related quality of life

**Fig. 2:**
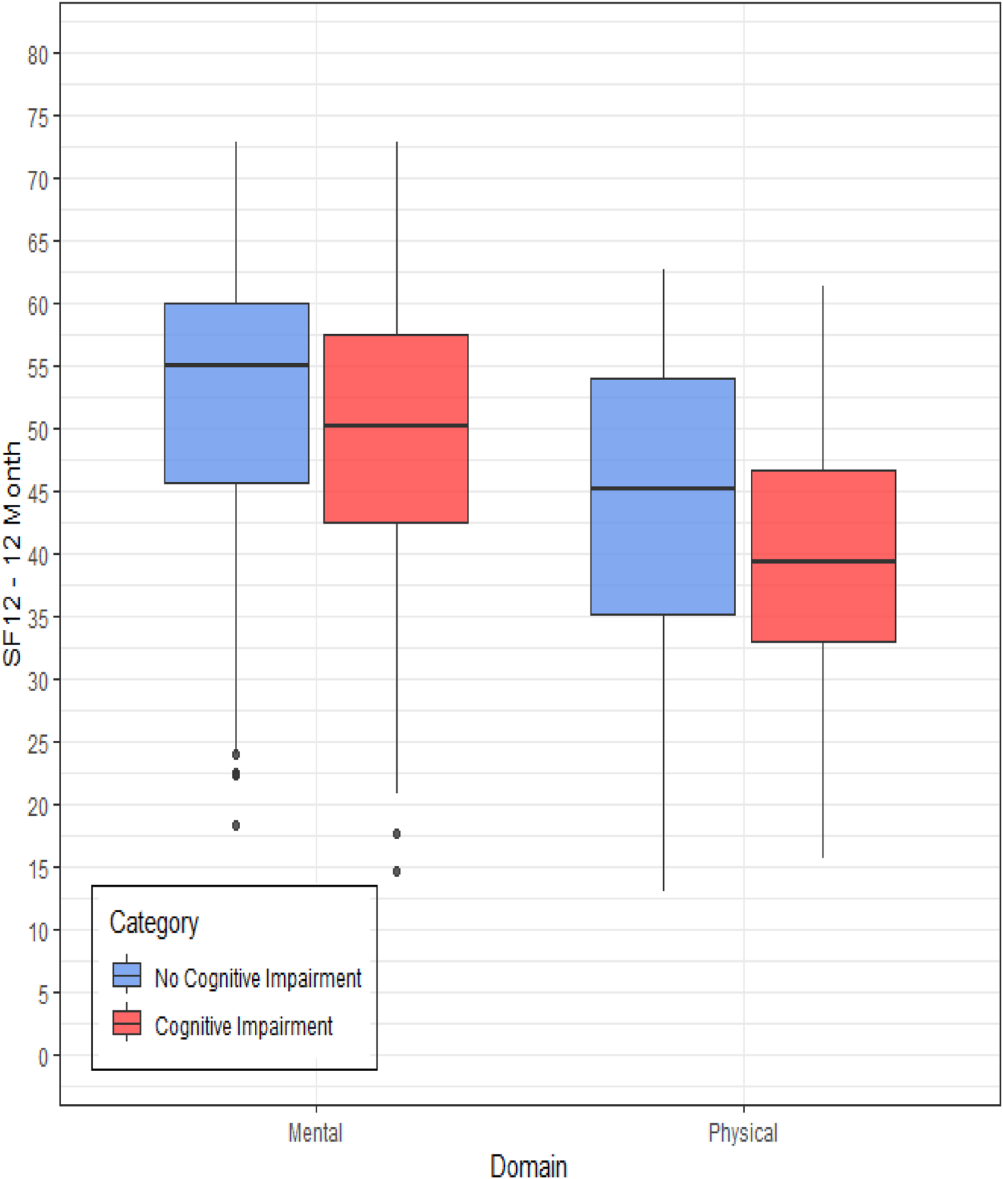
Bar graphs: comparison of 12-month quality-of-life scores among ICU survivors with or without cognitive dysfunction

The HRQoL assessed at 12 months by the mental dimension of the SF-12v2, was significantly better in patients who did not have cognitive dysfunction (Mean difference = − 2.54; CI 95%, − 4.80/− 0.28; p = 0.028). The physical dimension was also significantly better in patients who did not have cognitive dysfunction at 12 months (Mean difference = − 2.85; CI 95%, − 5.20/− 0.50; P = 0.018). (Fig. 2; Supplemental Table S3).

When analyzing the SF-12v2 domains, patients with cognitive dysfunction showed worse scores in the domains of general health, physical functioning, physical role function, bodily pain, vitality, mental health, and social functioning, but not in emotional role function (Supplemental Table S4).

When analyzing functional impairment, the study found no differences between patients with or without cognitive dysfunction at any time point (3mo: p = 0.09; 6mo: p = 0.07; 12mo: p = 0.33). (Supplemental Table S5).

## Discussion

Among the patients evaluated for cognitive dysfunction twelve months after ICU discharge, 351 (77.7%) had some degree of cognitive dysfunction (tMOCA < 16) and considering the study population with a tMOCA < 12, 216 (47.8%) patients had moderate to severe cognitive dysfunction. In the univariate analysis several factors were associated with an increased or decreased risk for cognitive dysfunction, but in the multivariate analysis only older age, delirium during ICU stay and pre-ICU high comorbidity were associated with an increased risk of having long-term cognitive dysfunction. Higher education was consistently protective against long-term cognitive dysfunction. The overall HRQoL assessed at 12 months was significantly better in patients who did not have cognitive dysfunction. Functional impairment and cognitive dysfunction were not associated throughout the natural history of ICU survivors.

The long-term cognitive dysfunction prevalence in our sample is worse than other studies conducted in high-resource/income contexts [8, 10, 34]. Still, we consider that it may be underestimated, both because in our study, we only considered moderate and severe cognitive dysfunction and also because the severity of functional or cognitive impairment may have prevented survivors to complete the assessment.

Results show an increase in cognitive dysfunction between discharge and 12 months follow up. The author interpretation is that the results found are secondary to the sensitivity of the chosen test and the use of MMSE at ICU discharge may have underestimated the true impact of early cognitive impairment, when compared to tMoCA or MoCA [8]. In accordance with previous published data [9] and clinical experience, author believe that the natural history of ICU acquired cognitive dysfunction is that almost all patients discharged from ICU experience different degree of cognitive dysfunction related to ICU admission and that it decreases throughout time. Authors decision to apply different tests was based on the assumption that the use of MoCa test at ICU discharge would result in a low response rate due to MoCa test complexity. Telephone assessment at 12 months did not allow the application of the MMSE in the same time frame.

We may interpret the prevalence of cognitive dysfunction found as a result of the historical economic evolution of Brazil, starting from a low-income country to becoming one of the largest economies in the world, but still having a middle class that represents 1/3 of the population [35]. In our cohort, the median household income per capita is comprised in the interval considered as middle-income class. Also, and, even though Brazil has, since 2009, one of the world’s longest duration of compulsory education, it is still paying the burden of years of non-investment and social discrepancies, as 1/4 of the population did not attend or finish high school, elementary school or are illiterate [35]. In our cohort, the average number of years of schooling, 11 years, suggests this reality. It is noteworthy that education level influenced the performance on tMOCA, sustainably as a protective factor. This may be explained through the concept of brain and cognitive reserve, that refers to the individuals ability to tolerate the age and disease-related changes without developing cognitive deterioration signs or symptoms [36, 37]. Our study evokes evidence of the importance of cognitive reserve, represented by a protective effect of higher education in the cognitive performance after ICU. The amount of cognitive reserve is likely acquired through educational attainment, physical and mental activity, occupational performance and successful social relationships. The association between higher household income and better cognitive outcome [38], may also be indicative of higher educational attainment.

Consistent with previous evidence, we found increasing age and comorbidity before ICU as associated factors for cognitive impairment [39–41]. Older age was consistently associated with worse cognitive outcome and may be a proxy for lower brain and cognitive reserve.

In a non-fragmented population of ICU survivors, and despite its low [6] incidence, the authors confirmed previous evidence that delirium is a predictor of worse cognitive outcome after ICU discharge [10, 42–44]. Delirium during ICU stay may as well be the only modifiable factor towards better cognitive outcome. When analyzing organ dysfunction, even though others dysfunctions have been previously implicated in long-term cognitive dysfunction [10, 45, 46], in our study only delirium was implicated in worse cognitive outcome. The dichotomized nature of the collected variables may have underestimated the true impact of acute organ dysfunction on long-term cognitive dysfunction.

Functional impairment after ICU depends on a myriad of causes and is currently described in the spectrum of ICU acquired weakness. In non-ICU patients, physical performance is an important predictor of cognitive performance [47]. In our cohort, muscle weakness early after ICU discharge was not statistically related to long-term cognitive dysfunction except in the sensitivity analysis. When analyzing functional impairment as a composite outcome, which include physical functioning, at 3 and 6 months, although it does not reach statistical significance, we can hypothesize that it may be clinically significant, and that only at 12 months does functional dependence loses a clear significance towards the cognitive outcome. This data is particularly relevant, as this points out to a possible modifiable associated factor for long-term cognitive dysfunction and for one of the possible early interventions during ICU admission that may improve long term outcome: early physical rehabilitation.

Quality of life measured by SF-12v2 is better for patients without cognitive dysfunction [4]. It can be hypothesized that quality of life after ICU depends on a prior construct and personal behaviors, with cognitive and brain reserve appearing to play a preemptive role in cognitive outcomes. If we think of brain and neural connections as something we can train, then the same is true for what should be the focus of rehabilitation after ICU: we should rehabilitate both the brain as well as the body [48, 49].

Potentially modifiable factors such as delirium and physical impairment must be screened during hospital stay. Early identification of ICU patients at increased risk for cognitive impairment, who might benefit from preventive measures or early rehabilitation, should be a future area of investment.

### Strengths and limitations

#### Strengths

The study has several major strengths: its prospective multicenter design, the inclusion of a large mixed population of ICU survivors, the prior definition of outcomes, the joint assessment of variables before, during and after ICU discharge in the same study and the use of standardized and validated instruments to measure cognitive outcomes [12]. Sensitivity analyses was performed to deal with possible bias.

#### Weaknesses

This study has several limitations. The study’s observational design may introduce inherent biases such as unmeasured confounders, selection bias, response and survival bias.

The study´s high sample size may have mitigated potential bias, but 59.2% of patients did not complete cognitive evaluation, potentially underestimated the prevalence and significance of the actual dysfunction. A significant limitation of the study is the large proportion of patients excluded for absence of cognitive evaluation as 12 months thus introducing a selection bias.

Another limitation is that tests used to evaluate cognitive function between discharge and 12 months follow-up were different making it difficult to understand the evolution of cognitive function. Also, the use of MMSE at ICU discharge may have underestimated the true impact of early cognitive impairment, when compared to tMoCA ou MoCA. Authors decision to apply different tests was based on the assumption that the use of MoCa test at ICU discharge would result in a low response rate due to MoCa test complexity.

In our cohort, for the purpose of a pragmatic study, except for dementia, we did not exclude patients with known medical diagnosis that might contribute to a worse cognitive outcome, and also potentially exacerbate the role of age as an associated factor.

Another limitation is the lack of pre-ICU cognitive assessment. Another study that assessed baseline cognitive status showed that in pre-ICU admission only 6% of patients had evidence of mild-to- moderate cognitive impairment [39, 50], suggesting that extrapolating pre-ICU cognitive dysfunction in our sample could yield similar results.

Finally, and even though psychological disorders can influence cognitive performance, the long-term outcomes specified in the study protocol [12] were planned to be mental illness at 6 month and cognitive dysfunction at 12 months. For that, authors cannot provide data concerning mental illness at 1 year.

## Conclusion

ICU-related cognitive impairment is a topic that requires continued attention, not only because cognitive function is a predictor of quality of life, but essentially because survivors won’t be able to return to their previous life if they do not recover their cognitive function.

The only clear potential modifiable associated factor for long-term cognitive dysfunction among ICU survivors was delirium. Higher education was consistently protective against long-term cognitive dysfunction, leading us to consider the importance of cognitive reserve and of the social and educational politics towards improving educational attainment. Regarding pre-ICU comorbidity, a call for action must take place towards a healthier lifestyle in order to decrease this potential modifiable associated factor.

The continuum of brain and physical dysfunction must be addressed from the outset. The importance of preventing and treating cognitive and physical dysfunction during ICU stay should be paralleled by rehabilitation after ICU discharge aiming to reduce ICU sequelae. Future clinical trials focusing on physical and cognitive rehabilitation could demonstrate the potential benefits of such interventions.

### Supplementary Information

Additional file 1.

## Data Availability

The datasets used and/or analyzed during the current study are available from the corresponding author on reasonable request.

## Abbreviations

APACHE-II: Acute Physiology and Chronic Health Evaluation II
ARDS: Acute respiratory distress syndrome
BI: Barthel Index
CAM-ICU: Confusion Assessment Method for the ICU
CCI: Charlson Comorbidity Index
CI: Confidence Interval
GEE: Generalized Estimative Equations
GLM: Generalized Linear Model
HADS-a: Hospital Anxiety and Depression Scale Anxiety
HADS-d: Hospital Anxiety and Depression Scale Depression
HRQoL: Health-Related Quality of Life
ICU: Intensive care unit
ICUAW: ICU acquired weakness
IQR: Interquartile range
ME: Mean difference
MMSE: Mini Mental State Examination
MRC: Medical Resuscitation Council Scale
PR: Prevalence Ratio
SAPS-3: Simplified Acute Physiology Score 3
SF-12v2: Short Form Survey: Short Form 12 Version 2
tMOCA: Telephone Montreal Cognitive Assessment
