# Supplementary material for: Long term cognitive dysfunction among critical care survivors: associated factors and quality of life—a multicenter cohort study
Source: Ann Intensive Care. 2024 Jul 29;14:116. doi: 10.1186/s13613-024-01335-w (PMC11286902; doi:10.1186/s13613-024-01335-w)
Supplement: Supplementary file 1 — Additional file 1. [file 13613_2024_1335_MOESM1_ESM.docx]

**Supplementary material**

**Long term cognitive dysfunction among Critical Care Survivors: associated factors and quality of life– A multicenter cohort study**

**Table of contents**

[List of Participant sites (by state and hospital center) 2](#_Toc160900065)

[Image 1 Participant flow chart diagram 3](#_Toc160900066)

[Table 1 Baseline characteristics of all patients with or without cognitive assessment at M12 4](#_Toc160900067)

[Table 2 Characteristics of Hospitals 7](#_Toc160900068)

[Table 3 Comparison of 12-month quality-of-life scores among ICU survivors with or without cognitive dysfunction 8](#_Toc160900069)

[Table 4 Comparison of 12-month quality-of-life subdomain scores among ICU survivors with or without cognitive dysfunction 9](#_Toc160900070)

[Table 6 Sensitivity analysis to assess the risk of survival bias 11](#_Toc160900071)

# List of Participant sites (by state and hospital center)

**Bahia**

*Hospital Geral Clériston Andrade* – Principal investigator: Lúcio Couto de Oliveira Júnior.

**Goiás**

*Hospital de Urgências de Goiânia* – Principal investigator: José Mario Meira Teles.

**Pará**

*Hospital Regional do Baixo Amazonas* – Principal investigator: Lívia Correa e Castro.

**Rio Grande do Sul**

*Hospital Ernesto Dornelles* – Principal investigator: André Sant’Ana Machado.

*Hospital de Clínicas de Porto Alegre* – Principal investigator: Silvia Regina Rios Vieira.

*Hospital Moinhos de Vento* – Principal investigator: Roselaine Pinheiro de Oliveira.

*Hospital Nossa Senhora da Conceição* – Principal investigator: Wagner Nedel.

*Hospital Santa Clara* – Principal investigator: Rodrigo Boldo.

*Pavilhão Pereira Filho* – Principal investigator: Daniella Cunha Birriel.

**São Paulo**

*Hospital do Coração* – Principal investigator: Alexandre Biasi Cavalcanti.

Image 1 Participant flow chart diagram

MOCA, Montreal cognitive assessment

Table 1 Baseline characteristics of all patients with or without cognitive assessment at M12

| **Sociodemographic characteristics** | **Completed assessment** | **Losses to follow-up** | **Death** |
| --- | --- | --- | --- |
| Age, years – median (IQR) | 60 (47.8-68.2) | 68 (52-78) | 69 (56-79) |
| Age ≥65 years – no./total no. (%) | 166/452 (36.7) | 373/656 (56.9) | 249/410 (60.7) |
| Female sex – no./total no. (%) | 215/452 (47.6) | 319/656 (48.6) | 186/410 (45.4) |
| Educational attainment, years – median (IQR) | 11 (8-16) | 9 (4-11) | 8 (4-11) |
| No Higher education – no./total no. (%) | 323/450 (71.8) | 526/655 (80.3) | 338/409 (82.6) |
| Monthly per capita household income^a^, USD – median (IQR) | 671.7 (403-1641.2) | 486.7 (249.5-1344.8) | 466.9 (268.9-806.9) |
| **State of health before admission to the ICU** |  |  |  |
| Charlson comorbidity index – median (IQR) | 1 (0-3) | 1 (0-3) | 3 (1-5) |
| Charlson comorbidity index ≥2 – no./total no. (%) | 209/452 (46.2) | 324/656 (49.4) | 293/410 (71.5) |
| History of depression – no./total no. (%) | 77/449 (17.1) | 138/651 (21.2) | 75/407 (18.4) |
| History of anxiety – no./total no. (%) | 88/451 (19.5) | 121/650 (18.6) | 69/407 (17) |
| Barthel Index – median (IQR) | 100 (95-100) | 100 (88.8-100) | 95 (75-100) |
| [0-25]-Total | 2/451 (0.4) | 27/656 (4.1) | 27/409 (6.6) |
| (25-50]-Severe | 7/451 (1.6) | 51/656 (7.8) | 62/409 (15.2) |
| (50-75]-Moderate | 13/451 (2.9) | 169/656 (25.8) | 137/409 (33.5) |
| (75-99]-Mild | 111/451 (24.6) | 33/656 (5) | 20/409 (4.9) |
| '100 - Independent | 318/451 (70.5) | 376/656 (57.3) | 163/409 (39.9) |
| **Characteristics of acute critical illness** |  |  |  |
| ICU Admission type |  |  |  |
| Medical – no./total no. (%) | 306/452 (67.7) | 443/656 (67.5) | 291/410 (71) |
| Surgical, elective – no./total no. (%) | 89/452 (19.7) | 114/656 (17.4) | 72/410 (17.6) |
| Surgical, emergency – no./total no. (%) | 57/452 (12.6) | 99/656 (15.1) | 47/410 (11.5) |
| Risk of death at ICU admission^b^, % – median (IQR) | 14.6 (8.7-26.2) | 18.6 (11.3-35.5) | 23.5 (14.1-52.3) |
| Severe sepsis or septic shock at ICU admission^c^ – no./total no. (%) | 152/452 (33.6) | 176/656 (26.8) | 149/410 (36.3) |
| ARDS at ICU admission^d^ – no./total no. (%) | 38/452 (8.4) | 25/656 (3.8) | 14/410 (3.4) |
| Organ dysfunction^e^ during ICU stay |  |  |  |
| Number of organ dysfunctions – median (IQR) | 1 (0-2) |  |  |
| Need of mechanical ventilation – no./total no. (%) | 215/452 (47.6) | 330/656 (50.3) | 201/410 (49) |
| Days of mechanical ventilation - median (IQR) | 0 (0-3) | 1 (0-4) | 0 (0-5) |
| Need of vasopressor – no./total no. (%) | 235/452 (52) | 335/656 (51.1) | 239/410 (58.3) |
| Need of renal replacement therapy – no./total no. (%) | 46/452 (10.2) | 80/656 (12.2) | 62/410 (15.1) |
| Need of parenteral nutrition – no./total no. (%) | 28/452 (6.2) | 36/656 (5.5) | 22/410 (5.4) |
| Need of blood or blood products transfusion – no./total no. (%) | 66/452 (14.6) | 119/656 (18.1) | 89/410 (21.7) |
| Delirium – no./total no. (%) | 90/452 (19.9) | 163/656 (24.8) | 118/410 (28.8) |
| ICU-acquired infection^f^ – no./total no. (%) | 48/452 (10.6) | 91/656 (13.9) | 70/410 (17.1) |
| ICU length of stay, days, median (IQR) | 6 (4-10) | 6 (5-11) | 7 (5-12) |
| Hospital length of stay, days, median (IQR) | 21 (13-36) | 24 (16-43.2) | 36 (22-55) |
| **State of health immediately after ICU discharge (24 to 120 hours)** |  |  |  |
| Respondents - HADS - no./total no. (%) | 412/452 (91.2) | 418/656 (63.7) | 232/410 (56.6) |
| HADS-a - median (IQR) | 6 (3-9.2) | 7 (4-10) | 6 (3-9) |
| Anxiety symptoms^g^ (HADSa >7) - no./total no. (%) | 161/412 (39.1) | 175/418 (41.9) | 94/232 (40.5) |
| HADS-d - median (IQR) | 4 (2-7) | 5 (2-8) | 5 (3-9) |
| Depression symptoms^h^ (HADSd >7) - no./total no. (%) | 89/412 (21.6) | 121/418 (28.9) | 73/232 (31.5) |
| Respondents – MMSE^i^- no./total no. (%) | 363/452 (80.3) | 400/656 (61) | 219/410 (53.4) |
| MMSE score - median (IQR) | 25 (23-27) | 23 (19-27) | 23 (19-26) |
| Cognitive dysfunction - no./total no. (%) | 118/363 (32.5) | 198/400 (49.5) | 118/219 (53.9) |
| Respondents - MRC - no./total no. (%) | 322/452 (71.2) | 404/656 (61.6) | 229/410 (55.9) |
| MRC - median (IQR) | 55 (48-60) | 51 (45-60) | 48 (43-57) |
| Muscular weakness (MCR <48) - no./total no. (%) | 68/322 (21.1) | 126/404 (31.2) | 88/229 (38.4) |

IQR, interquartile range (p25; p75); ICU, intensive care unit; ARDS, cute respiratory distress syndrome; HADS, hospital anxiety and depression scale; MMSE, mini-mental state evaluation; MRC, medical research council scale

^a^ USD, United States dollar using the purchasing power parity conversion (BRL to USD);

^b^ Predicted risk of hospital death derived from the Acute Physiology and Chronic Health Evaluation-II (APACHE-II) or the Simplified Acute Physiology Score-3 (SAPS-3);

^c^ According to the sepsis-II criteria;

^d^ According to Berlin criteria;

^e^ Defined as the presence of any of the following during ICU stay: need of invasive mechanical ventilation, vasopressor, renal replacement therapy (except for patients under chronic dialysis treatment), parenteral nutrition, blood or blood products transfusion; and delirium (measured according the Confusion Assessment Method for the ICU);

^f^ Defined as pneumonia, bloodstream or urinary tract infection occurring > 48 hours of ICU admission according to the European Centre for Disease Prevention and Control criteria

^g^ Anxiety Hospital Anxiety and Depression Scale anxiety subscale score > 7.

^h^ Anxiety Hospital Anxiety and Depression Scale depression subscale score > 7.

^I^ Mini Mental State Examination ≤21 if 4 years or less of educational attainment, or ≤24 if >4 years of educational attainment.

Table 2 Characteristics of Hospitals

| **Characteristics** | **Values** |
| --- | --- |
| **Hospitals, *n* = 10** |  |
| Public—*n*/total *n* (%) | 5/10 (50.0) |
| Private—*n* /total *n* (%) | 5/10 (50.0) |
| Academic—*n*/total *n* (%) | 3/10 (30.0) |
| Number of hospital beds—median (IQR) (*n* assessed) | 371.0 (292.0–670.0) (10) |
| Number of ICU beds—median (IQR) (*n* assessed) | 20.0 (20.0–30.0) (10) |
| ICU-to-hospital bed ratio, % (IQR) (*n* assessed] | 6.6 (5.1–8.0) (10) |

IQR, interquartile range (p25; p75); ICU, intensive care unit.

Table 3 Comparison of 12-month quality-of-life scores among ICU survivors with or without cognitive dysfunction

|  | **Total** | **No Cognitive Dysfunction** | **Cognitive Dysfunction** | **Mean Difference (ME) (95% CI)** | **P value** |
| --- | --- | --- | --- | --- | --- |
| SF12 - 12 Month |  |  |  |  |  |
| Physical | 42.0 (11.0) | 43.7 (11.4) | 39.9 (10.1) | -2.54 (-4.80;-0.28) | 0.028 |
| Mental | 50.9 (11.2) | 52.1 (10.9) | 49.5 (11.4) | -2.85 (-5.20;-0.50) | 0.018 |

ME, Mean difference; SF12, Short Form Health Survey

Table 4 Comparison of 12-month quality-of-life subdomain scores among ICU survivors with or without cognitive dysfunction

|  | **Total** | **No Cognitive Dysfunction** | **Cognitive Dysfunction** | **Mean Difference (ME) (95% CI)** | **P value** |
| --- | --- | --- | --- | --- | --- |
| Physical functioning | 56.4 (35.6) | 61.9 (36.2) | 50.3 (33.9) | -8.65 (-15.72; -1.57) | 0.017 |
| Physical role function | 66.2 (28) | 69.9 (28.9) | 61.9 (26.2) | -6.64 (-12.43; -0.86) | 0.024 |
| Bodily pain | 70.5 (29.5) | 74.7 (28.4) | 65.7 (30.1) | -7.21 (-13.33;-1.09) | 0.021 |
| General health | 56.8 (28.1) | 62.1 (27.9) | 50.9 (27.2) | -9.57 (-15.24; -3.90) | 0.001 |
| Vitality | 58.2 (28.5) | 60.7 (26) | 55.2 (31.1) | -6.54 ( -12.55; -0.54) | 0.033 |
| Social functioning | 70.7 (32.5) | 75 (32) | 65.7 (32.5) | -8.87 (-15.66; -2.08) | 0.001 |
| Emotional role function | 80.2 (23.9) | 82.8 (22.2) | 77.2 (25.4) | -3.88 (-8.83; 1.07) | 0.125 |
| Mental health | 70.4 (23.8) | 73.1 (22.8) | 67.3 (24.6) | -6.30 (-11.24; -1.36) | 0.012 |

ME, Mean difference

Table 5 Functional impairment at 3, 6 and 12 months after ICU discharge

| **Functional impairment** | **Total** | **No Cognitive Disfunction** | **Cognitive Disfunction** | **P-value** |
| --- | --- | --- | --- | --- |
| 3 Months | 74/408 (18.1) | 31/210 (14.8) | 43/198 (21.7) | 0.09 |
| 6 Months | 43/423 (10.2) | 16/218 (7.3) | 27/205 (13.2) | 0.07 |
| 12 Months | 33/451 (7.3) | 14/235 (6) | 19/216 (8.8) | 0.33 |

Table 6 Sensitivity analysis to assess the risk of survival bias

|  | **Total** | **No Cognitive Dysfunction** | **Cognitive Dysfunction or Death** |  | |
| --- | --- | --- | --- | --- | --- |
|  |  |  |  | Prevalence ratio  (PR) (95% CI) | *P Value* |
| **Sociodemographic characteristics** |  |  |  |  |  |
| Age, years – median (IQR) | 63 (51-74) | 57 (41-66.2) | 66 (56-77) | 1.01 (1.01 - 1.01) | <0.001 |
| Age >=65 years – no./total no. (%) | 415/862 (48.1) | 73/236 (30.9) | 342/626 (54.6) | 1.3 (1.19 - 1.41) | <0.001 |
| Female sex – no./total no. (%) | 401/862 (46.5) | 118/236 (50) | 283/626 (45.2) | 0.95 (0.87 - 1.03) | 0.211 |
| Educational attainment, years – median (IQR) | 11 (5-11) | 11 (11-16) | 8 (4-11) | 0.95 (0.95 - 0.96) | <0.001 |
| No higher education – no./total no. (%) | 661/859 (76.9) | 135/236 (57.2) | 526/623 (84.4) | 1.62 (1.4 - 1.88) | <0.001 |
| Monthly per capita household income^a^, USD – median (IQR) | 537.9 (324.5-1259.4) | 1175.5 (494.1-2686.8) | 484.2 (292-849.8) | 1 (1 - 1) | 0.022 |
| **State of health before admission to the ICU** |  |  |  |  |  |
| Charlson comorbidity index – median (IQR) | 2 (0-4) | 1 (0-2) | 2 (1-4) | 1.06 (1.04 - 1.08) | <0.001 |
| Charlson comorbidity index >=2 – no./total no. (%) | 502/862 (58.2) | 93/236 (39.4) | 409/626 (65.3) | 1.35 (1.23 - 1.48) | <0.001 |
| History of depression – no./total no. (%) | 152/856 (17.8) | 42/235 (17.9) | 110/621 (17.7) | 1 (0.89 - 1.11) | 0.957 |
| History of anxiety – no./total no. (%) | 157/858 (18.3) | 51/236 (21.6) | 106/622 (17) | 0.92 (0.82 - 1.03) | 0.148 |
| Barthel Index – median (IQR) | 100 (90-100) | 100 (95-100) | 95 (85-100) |  | <0.001 |
| [0-25]-Total | 22/860 (2.6) | 1/235 (0.4) | 21/625 (3.4) |  |  |
| (25-50]-Severe | 34/860 (4) | 4/235 (1.7) | 30/625 (4.8) |  |  |
| (50-75]-Moderate | 75/860 (8.7) | 7/235 (3) | 68/625 (10.9) |  |  |
| (75-99]-Mild | 248/860 (28.8) | 54/235 (23) | 194/625 (31) |  |  |
| '100 - Independent | 481/860 (55.9) | 169/235 (71.9) | 312/625 (49.9) |  |  |
| **Characteristics of acute critical illness** |  |  |  |  |  |
| ICU Admission type |  |  |  |  | 0.592 |
| Medical – no./total no. (%) | 597/862 (69.3) | 160/236 (67.8) | 437/626 (69.8) |  |  |
| Surgical, elective – no./total no. (%) | 161/862 (18.7) | 46/236 (19.5) | 115/626 (18.4) |  |  |
| Surgical, emergency – no./total no. (%) | 104/862 (12.1) | 30/236 (12.7) | 74/626 (11.8) |  |  |
| Risk of death at ICU admission^b^, % – median (IQR) | 18.6 (11.3-35.5) | 14.6 (8.7-23.5) | 21 (12.9-42.9) | 1.01 (1 - 1.01) | <0.001 |
| Severe sepsis or septic shock at ICU admission^c^ – no./total no. (%) | 301/862 (34.9) | 74/236 (31.4) | 227/626 (36.3) | 1.06 (0.98 - 1.15) | 0.168 |
| ARDS at ICU admission^d^ – no./total no. (%) | 52/862 (6) | 22/236 (9.3) | 30/626 (4.8) | 0.78 (0.62 - 0.99) | 0.044 |
| Organ dysfunction^e^ during ICU stay |  |  |  |  |  |
| Number of organ dysfunctions – median (IQR) |  |  |  |  |  |
| Need of mechanical ventilation – no./total no. (%) | 416/862 (48.3) | 117/236 (49.6) | 299/626 (47.8) | 0.98 (0.9 - 1.06) | 0.635 |
| Days of mechanical ventilation - median (IQR) | 0 (0-4) | 0 (0-4) | 0 (0-4) | 1 (1 - 1.01) | 0.044 |
| Need of vasopressor – no./total no. (%) | 474/862 (55) | 128/236 (54.2) | 346/626 (55.3) | 1.01 (0.93 - 1.1) | 0.786 |
| Need of renal replacement therapy – no./total no. (%) | 108/862 (12.5) | 25/236 (10.6) | 83/626 (13.3) | 1.07 (0.95 - 1.19) | 0.258 |
| Need of parenteral nutrition – no./total no. (%) | 50/862 (5.8) | 11/236 (4.7) | 39/626 (6.2) | 1.08 (0.93 - 1.26) | 0.331 |
| Need of blood or blood products transfusion – no./total no. (%) | 155/862 (18) | 37/236 (15.7) | 118/626 (18.8) | 1.06 (0.96 - 1.17) | 0.255 |
| Delirium – no./total no. (%) | 208/862 (24.1) | 38/236 (16.1) | 170/626 (27.2) | 1.17 (1.08 - 1.27) | <0.001 |
| ICU-acquired infection^f^ – no./total no. (%) | 118/862 (13.7) | 29/236 (12.3) | 89/626 (14.2) | 1.04 (0.93 - 1.17) | 0.442 |
| ICU length of stay, days, median (IQR) | 7 (4-11) | 7 (4-11) | 7 (4-10.8) | 1 (1 - 1) | 0.429 |
| Hospital length of stay, days, median (IQR) | 27 (16-47) | 21 (14-38.2) | 30 (18-49) | 1 (1 - 1) | 0.022 |
| **State of health immediately after ICU discharge (24 to 120 hours)** |  |  |  |  |  |
| Respondents - HADS - no./total no. (%) | 644/862 (74.7) | 214/236 (90.7) | 430/626 (68.7) | 0.74 (0.69 - 0.8) | <0.001 |
| HADS-a - median (IQR) | 6 (3-9) | 5.5 (3-9) | 7 (3-10) | 1.02 (1 - 1.03) | 0.009 |
| Anxiety symptoms^g^ (HADSa >7) - no./total no. (%) | 255/644 (39.6) | 70/214 (32.7) | 185/430 (43) | 1.15 (1.03 - 1.28) | 0.01 |
| HADS-d - median (IQR) | 4 (3-8) | 4 (2-7) | 5 (3-8) | 1.03 (1.01 - 1.04) | <0.001 |
| Depression symptoms^h^ (HADSd >7) - no./total no. (%) | 162/644 (25.2) | 42/214 (19.6) | 120/430 (27.9) | 1.15 (1.03 - 1.29) | 0.014 |
| Respondents – MMSE^i^-no./total no. (%) | 582/862 (67.5) | 187/236 (79.2) | 395/626 (63.1) | 0.82 (0.76 - 0.89) | <0.001 |
| MMSE score - median (IQR) | 24 (22-27) | 27 (24-29) | 24 (20-26) | 0.96 (0.96 - 0.97) | <0.001 |
| Cognitive dysfunction - no./total no. (%) | 236/582 (40.5) | 48/187 (25.7) | 188/395 (47.6) | 1.33 (1.2 - 1.48) | <0.001 |
| Respondents - MRC - no./total no. (%) | 551/862 (63.9) | 158/236 (66.9) | 393/626 (62.8) | 0.95 (0.88 - 1.03) | 0.247 |
| MRC - median (IQR) | 52 (46-60) | 55.5 (48-60) | 50 (44-59) | 0.99 (0.99 - 1) | 0.013 |
| Muscular weakness (MCR <48) - no./total no. (%) | 156/551 (28.3) | 34/158 (21.5) | 122/393 (31) | 1.14 (1.02 - 1.27) | 0.016 |

IQR, interquartile range (p25; p75); ICU, intensive care unit; ARDS, cute respiratory distress syndrome; HADS, hospital anxiety and depression scale; MMSE, mini-mental state evaluation; MRC, medical research council scale

^a^ USD, United States dollar using the purchasing power parity conversion (BRL to USD);

^b^ Predicted risk of hospital death derived from the Acute Physiology and Chronic Health Evaluation-II (APACHE-II) or the Simplified Acute Physiology Score-3 (SAPS-3);

^c^ According to the sepsis-II criteria;

^d^ According to Berlin criteria;

^e^ Defined as the presence of any of the following during ICU stay: need of invasive mechanical ventilation, vasopressor, renal replacement therapy (except for patients under chronic dialysis treatment), parenteral nutrition, blood or blood products transfusion; and delirium (measured according the Confusion Assessment Method for the ICU);

^f^ Defined as pneumonia, bloodstream or urinary tract infection occurring > 48 hours of ICU admission according to the European Centre for Disease Prevention and Control criteria

^g^ Anxiety Hospital Anxiety and Depression Scale anxiety subscale score > 7.

^h^ Anxiety Hospital Anxiety and Depression Scale depression subscale score > 7.

^I^ Mini Mental State Examination ≤21 if 4 years or less of educational attainment, or ≤24 if >4 years of educational attainment.
